# Uncovering the sex steroid hormone secrets in alcohol

**DOI:** 10.1111/acer.15479

**Published:** 2024-11-10

**Authors:** Gian Rodriguez Franco, Christine C. Hsu

**Affiliations:** ^1^ Liver Disease Branch National Institute of Diabetes and Digestive and Kidney Diseases Bethesda Maryland USA

Alcohol use disorder (AUD) is a significant global health problem, affecting millions of individuals and resulting in substantial economic, social, and health‐related burdens (Griswold et al., 2018; Sacks et al., 2015). Alcohol has contributed to an estimated 3.8%–5.3% of all global deaths and in 2016 it resulted in three million deaths (Global status report on alcohol and health 2018, 2018; Rehm et al., 2009). Chronic alcohol consumption has been associated with a wide array of adverse health outcomes, including liver cirrhosis, cardiovascular diseases, pancreatitis, various cancers, and neurological disorders (Sterling et al., 2020). The biochemical mechanisms that are responsible for the adverse health effects are not fully understood and different modalities are recently being used to understand these (Voutilainen & Kärkkäinen, 2019). Metabolomics, which is the comprehensive study of metabolites or small molecules involved in complex biochemical reactions in: cells, tissues, or biofluids can be an important tool to provide insight into pathophysiological processes and cellular changes in humans and has the potential to lead to the identification of biomarkers that can aid in early diagnosis or guide in treatment responses (Joshi et al., 2023). Approaches can either be targeted (measurement of prespecified metabolites) or untargeted and techniques used include nuclear magnetic resonance (NMR) spectroscopy or mass spectrometry (MS) (Joshi et al., 2023). Metabolomics has been previously explored in alcoholic liver disease (ALD) and in heavy alcohol drinkers. One study showed that urine metabolites involved in caffeine metabolism are significantly decreased in ALD patients compared with controls, correlating with the severity of liver disease (MELD) (Xu, He, et al., 2023). Other studies have demonstrated that pathways involved in bile acid and amino acid metabolism are altered in patients with either ALD or alcohol liver cirrhosis (Xu, Hao, et al., 2023; Xu, Vatsalya, et al., 2023). One identified metabolite, *N*‐Luaroglycine, has been shown to have 100% sensitivity and 90% negative predictive value in identifying cirrhosis in ALD patients (Suciu et al., 2018); however, most studies have been limited to small numbers of patients and have yet to be validated in large cohorts of patients. The largest study to date is from Japan, where the authors analyzed the plasma of male chronic alcohol drinkers (*n* = 896) (Harada et al., 2016). They identified 19 metabolites (involved in amino acid, carbohydrate, lipid, and vitamin metabolism) that correlated with alcohol consumption and increased threonine and decreased levels of guanidinosuccinate and glutamine were associated with alcohol‐induced liver injury (Harada et al., ).

We read with interest the recent *ACER* manuscript by Yang et al. (2024) as they further explore the unique metabolomic signatures in urine and serum of patients with excessive alcohol use and discuss their findings in this commentary. Conducted as an exploratory study, it involved 22 healthy controls and 38 patients identified with excessive alcohol consumption defined by NIAAA criteria as ≥4 standard drinks per day (≥14/week) for men and ≥3 standard drinks per day (≥7/week) for women. The authors identified patients through AUDIT‐C and Timeline Follow Back questionnaires. The authors utilized LC–MS/MS (liquid chromatography with tandem mass spectroscopy) to identify significant alterations in metabolic pathways, such as lipid metabolism, amino acid and peptide metabolism, cofactors and vitamin metabolism, carbohydrate metabolism, and nucleotide metabolism. Notably, 5α‐Androstan‐3β,17β‐diol disulfate, and androstenediol (3beta, 17beta) disulfate, both steroid hormones, were elevated in both urine and serum samples of excessive drinkers (Yang et al., 2024).

The results demonstrated substantial differences in metabolite profiles among the two groups. In urine samples, they identified 131 increased and 45 decreased compounds of excessive drinkers compared with controls, while in serum samples, 195 increased and 73 decreased compounds were identified among excessive drinkers compared with controls. The main metabolic pathways affected was lipid metabolism in both urine and serum samples, which is not surprising as changes in lipid metabolism have been found to be crucial in the pathogenesis of alcohol‐induced steatosis (Voutilainen & Kärkkäinen, 2019). Interestingly, only five metabolites were significantly altered in both urine and serum samples of excessive drinkers. Among these, only the steroid hormones serum 5α‐Androstan‐3β,17β‐diol disulfate and androstenediol (3beta, 17beta) disulfate were significantly elevated in both biofluids, suggesting their potential as biomarkers.

The study's design and results are noteworthy for multiple reasons. The study addresses the systemic impact of alcohol on multiple organ systems providing a comprehensive analysis of both urine and serum metabolomes and showed elevations in both urine and serum 5α‐Androstan‐3β,17β‐diol disulfate and androstenediol (3beta, 17beta) disulfate and its rigor is evidenced by the sample preparation and metabolomic analysis ensuring accurate comparisons by normalizing metabolite levels based on urine osmolality. The utilization of principal component analysis (PCA) and partial least square discriminant analysis (PLS‐DA) analyses further strengthens the study, revealing a clear segregation of metabolites between excessive drinkers and controls.

The study has limitations. The relatively small sample size and the lack of more specific methods to determine underlying liver disease, such as vibration‐controlled transient elastography or liver biopsy, limit the study's ability to provide definitive conclusions. While they discovered that serum level of androstenediol (3beta, 17beta) disulfate are higher in those with FIB‐4 ≥2.67, than in those with FIB‐4 <1.3, it has been shown that FIB‐4 has high false positive rate (35%) in an at‐risk population for alcohol‐associated liver disease (ALD) or metabolic dysfunction‐associated steatotic liver disease (Kjaergaard et al., 2023). This may suggest that while there is an association between elevated serum levels of sex hormone metabolites with elevated FIB‐4 scores, it may not be highly accurate in detecting advanced fibrosis or cirrhosis given the limitations of FIB‐4. However, there is a paucity of biomarkers that predict early ALD with accuracy, and thus, sex hormone metabolites should be further explored as a biomarker of early disease. The study presents a snapshot of metabolite levels at the time of enrollment. To gain a better understanding of the changes associated with alcohol use and to confirm the utility of identified biomarkers, longitudinal studies and the trajectory of the metabolite levels would be of value to confirm the usefulness of these biomarkers. Additionally, monitoring of metabolite levels after alcohol cessation, could help identify the dynamic nature of these alterations. By analyzing a broader range of sample types, such as tissue samples, deeper insight into the systemic effects of alcohol on the body could also be feasible. Lastly, combining metabolomics with other “omics” approaches, such as proteomics and transcriptomics, could help provide a more integrated understanding of the molecular changes induced by alcohol.

5α‐Androstan‐3β,17β‐diol disulfate is a sulfated metabolite of 5α‐androstane‐3β,17β‐diol, while androstenediol (3β,17β) is a precursor to androstenediol (3β,17β) disulfate (Handa et al., 2011). They are both endogenous steroid hormones; 5α‐androstane‐3β,17β‐diol is a principal metabolite of dihydrotestosterone (DHT), while androstenediol is an intermediary in the synthesis of testosterone from dehydroepiandrosterone (Figure 1) (Handa et al., 2011). Alcohol consumption has been shown to disrupt the hypothalamic–pituitary–gonadal axis, which regulates the production and conversion of sex hormones (Rachdaoui & Sarkar, 2017); however, it is unclear exactly where alcohol impacts the testosterone synthetic pathway. Studies have also shown that alcohol has a detrimental effect on Leydig cells which are responsible for steroidogenesis from increased oxidative stress (Maneesh et al., 2006). Similar to Yang et al., sulfated steroid hormones have been associated with alcohol intake in past studies (Guertin et al., 2014; Pallister et al., 2016). Both Guertin et al. and Palister et al. identified both 5α‐androstane‐3β,17β‐diol disulfate and 4‐Androsten‐3β,17β‐diol disulfate 1 to be associated with alcohol intake (Guertin et al., 2014; Pallister et al., 2016). Pallister et al. then further showed these metabolites were associated with a variant gene *SULT2A1*, which is responsible for sulfation of variety of steroids and bile acids (Pallister et al., 2016). Maiti et al. corroborated in animal studies where ethanol feeding in rats significantly increased liver and intestinal expression of *SULT2A1* suggesting a role for this gene in modulating the association (Maiti & Chen, 2015). Sulfation, facilitated by sulfotransferases (SULTs), increases the solubility of the steroid hormone, alters its biological activity and physiological processes, which affects interactions with steroid hormone receptors, or impacts androgen or estrogen activity (Mueller et al., 2015). SULTs play an important role in phase II drug metabolism and can be involved in the biotransformation of molecules to less lipophilic and more water‐soluble to allow for quicker elimination (Xie & Xie, 2020). SULTs also facilitate the sulfonation of a variety of substrates, including hormones, neurotransmitters, bile acids, and xenobiotics (Mrdjen et al., 2023; Xie & Xie, 2020). Among the SULTs, SULT2A1 is involved in the metabolism of steroid hormones and bile acids (Mrdjen et al., 2023). The expression of SULT2A1, along with other key sulfotransferases such as SULT1A1 and SULT1E1, has now been shown to be dysregulated in the context of alcohol‐associated liver disease (ALD), particularly in patients with severe alcohol‐associated hepatitis (sAH) (Mrdjen et al., 2023). In the study by Mrdjen et al., hepatic expression of SULT2A1 was significantly decreased in patients with forms of severe ALD, as evidenced by RNA sequencing and protein expression analysis (Mrdjen et al., 2023). This reduction in SULT2A1 expression is consistent with the dysregulation observed in phase II metabolic pathways among these patients. The decreased expression of SULT2A1 may contribute to the impaired metabolism of steroids and bile acids, which could potentially exacerbate the pathological effects of chronic alcohol consumption on the liver. Interestingly, it has been previously shown that females with alcohol dependence have higher levels of testosterone than those without alcohol dependence and males with alcohol dependence have higher estradiol (testosterone is a precursor to estradiol; Figure 1) and sex hormone binding globulin levels than those without. Thus, potentially, the downregulation of key sulfotransferases such as SULT2A1 and SULT1A1 can lead to decreased metabolism, which exacerbates underlying steatotic liver disease. However, while the expression of key sulfotransferases like SULT2A1 and SULT1A1 was downregulated, some less highly expressed sulfotransferases were found to be upregulated in patients with sAH (Mrdjen et al., 2023). This differential expression pattern suggests a compensatory mechanism, although the specific implications of increased expression of these other SULT enzymes remain unclear. Additional research is warranted to clarify the clinical implications of these findings and how the altered expression of sulfotransferases in ALD could become a target for therapeutic intervention.

**FIGURE 1:**
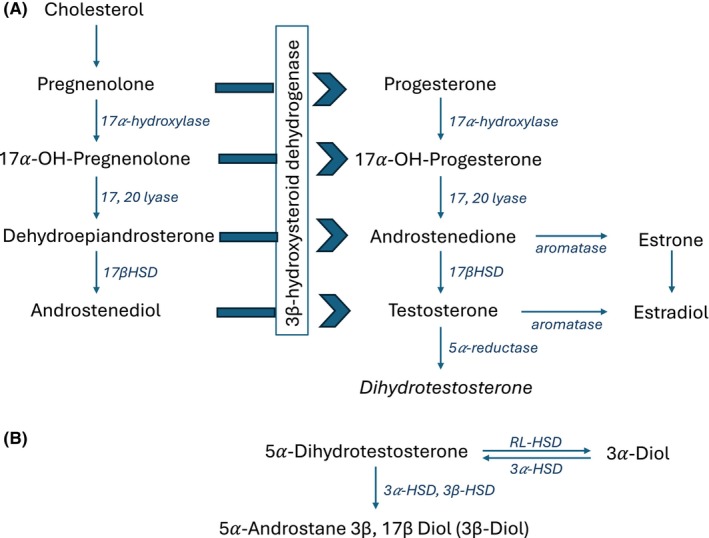
Synthetic pathway for gonadal steroid hormones. (A) Shows known enzymes and intermediates involved in synthesizing estrogens and androgens from cholesterol. (B) Demonstrates the steps of dihydrotestosterone processing, which are potentially involved in prereceptor regulation. 3α‐Diol = 5α androstane 3α, 17β Diol; 3β Diol = 5α androstane 3β, 17β Diol, RL‐HSD = 11‐cis‐retinol dehydrogenase like 3α‐HSD. Adapted from: Handa et al. (2011).

In conclusion, the study by Yang et al. reveals a notable association between sex hormone metabolites and excessive alcohol consumption in a pilot study. The identification of these specific metabolites as potential biomarkers for alcohol use and even alcohol‐associated liver disease emphasizes the prospect of metabolomics in the early detection of AUD and ALD. However, further research is needed to confirm these findings in a larger cohort of patients and in patients with established ALD to better understand the clinical implications of these metabolic changes. Additionally, exploring the underlying mechanisms by which alcohol alters metabolic pathways could provide deeper insights into the pathogenesis of alcohol‐related diseases and potentially unveil new therapeutic targets for intervention.

## CONFLICT OF INTEREST STATEMENT

The authors declare no conflicts of interest.

## Data Availability

Data sharing not applicable—no new data generated.

## References

[acer15479-bib-0001] (2018) Global status report on alcohol and health 2018. Geneva: World Health Organization. Available from: https://www.who.int/publications/i/item/9789241565639 [Accessed 15th October 2024].

[acer15479-bib-0002] Griswold, M.G. , Fullman, N. , Hawley, C. , Arian, N. , Zimsen, S.R.M. , Tymeson, H.D. et al. (2018) Alcohol use and burden for 195 countries and territories, 1990–2016: a systematic analysis for the global burden of disease study 2016. Lancet, 392(10152), 1015–1035. Available from: 10.1016/S0140-6736(18)31310-2 30146330 PMC6148333

[acer15479-bib-0003] Guertin, K.A. , Moore, S.C. , Sampson, J.N. , Huang, W.Y. , Xiao, Q. , Stolzenberg‐Solomon, R.Z. et al. (2014) Metabolomics in nutritional epidemiology: identifying metabolites associated with diet and quantifying their potential to uncover diet‐disease relations in populations. American Journal of Clinical Nutrition, 100(1), 208–217. Available from: 10.3945/ajcn.113.078758 24740205 PMC4144099

[acer15479-bib-0004] Handa, R.J. , Sharma, D. & Uht, R.M. (2011) A role for the androgen metabolite, 5alpha androstane 3beta, 17beta diol (3β‐diol) in the regulation of the Hypothalamo‐pituitary–adrenal Axis. Frontiers in Endocrinology, 2, 65. Available from: 10.3389/fendo.2011.00065 22649380 PMC3355903

[acer15479-bib-0005] Harada, S. , Takebayashi, T. , Kurihara, A. , Akiyama, M. , Suzuki, A. , Hatakeyama, Y. et al. (2016) Metabolomic profiling reveals novel biomarkers of alcohol intake and alcohol‐induced liver injury in community‐dwelling men. Environmental Health and Preventive Medicine, 21(1), 18–26. Available from: 10.1007/s12199-015-0494-y 26459263 PMC4693765

[acer15479-bib-0006] Joshi, A.D. , Rahnavard, A. , Kachroo, P. , Mendez, K.M. , Lawrence, W. , Julian‐Serrano, S. et al. (2023) An epidemiological introduction to human metabolomic investigations. Trends in Endocrinology and Metabolism, 34(9), 505–525. Available from: 10.1016/j.tem.2023.06.006 37468430 PMC10527234

[acer15479-bib-0007] Kjaergaard, M. , Lindvig, K.P. , Thorhauge, K.H. , Andersen, P. , Hansen, J.K. , Kastrup, N. et al. (2023) Using the ELF test, FIB‐4 and NAFLD fibrosis score to screen the population for liver disease. Journal of Hepatology, 79(2), 277–286. Available from: 10.1016/j.jhep.2023.04.002 37088311

[acer15479-bib-0008] Maiti, S. & Chen, G. (2015) Ethanol up‐regulates phenol sulfotransferase (SULT1A1) and hydroxysteroid sulfotransferase (SULT2A1) in rat liver and intestine. Archives of Physiology and Biochemistry, 121(2), 68–74. Available from: 10.3109/13813455.2014.992440 25720860

[acer15479-bib-0009] Maneesh, M. , Dutta, S. , Chakrabarti, A. & Vasudevan, D.M. (2006) Alcohol abuse‐duration dependent decrease in plasma testosterone and antioxidants in males. Indian Journal of Physiology and Pharmacology, 50(3), 291–296. 17193902

[acer15479-bib-0010] Mrdjen, M. , Huang, E. , Pathak, V. , Bellar, A. , Welch, N. , Dasarathy, J. et al. (2023) Dysregulated meta‐organismal metabolism of aromatic amino acids in alcohol‐associated liver disease. Hepatology Communications, 7(11), e0284. Available from: 10.1097/HC9.0000000000000284 37820283 PMC10578770

[acer15479-bib-0011] Mueller, J.W. , Gilligan, L.C. , Idkowiak, J. , Arlt, W. & Foster, P.A. (2015) The regulation of steroid action by sulfation and desulfation. Endocrine Reviews, 36(5), 526–563. Available from: 10.1210/er.2015-1036 26213785 PMC4591525

[acer15479-bib-0012] Pallister, T. , Jennings, A. , Mohney, R.P. , Yarand, D. , Mangino, M. , Cassidy, A. et al. (2016) Characterizing blood metabolomics profiles associated with self‐reported food intakes in female twins. PLoS One, 11(6), e0158568. Available from: 10.1371/journal.pone.0158568 27355821 PMC4927065

[acer15479-bib-0013] Rachdaoui, N. & Sarkar, D.K. (2017) Pathophysiology of the effects of alcohol abuse on the endocrine system. Alcohol Research: Current Reviews, 38(2), 255–276. 28988577 10.35946/arcr.v38.2.08 PMC5513689

[acer15479-bib-0014] Rehm, J. , Mathers, C. , Popova, S. , Thavorncharoensap, M. , Teerawattananon, Y. & Patra, J. (2009) Global burden of disease and injury and economic cost attributable to alcohol use and alcohol‐use disorders. Lancet, 373(9682), 2223–2233. Available from: 10.1016/S0140-6736(09)60746-7 19560604

[acer15479-bib-0015] Sacks, J.J. , Gonzales, K.R. , Bouchery, E.E. , Tomedi, L.E. & Brewer, R.D. (2015) 2010 national and state costs of excessive alcohol consumption. American Journal of Preventive Medicine, 49(5), e73–e79. Available from: 10.1016/j.amepre.2015.05.031 26477807

[acer15479-bib-0016] Sterling, S.A. , Palzes, V.A. , Lu, Y. , Kline‐Simon, A.H. , Parthasarathy, S. , Ross, T. et al. (2020) Associations between medical conditions and alcohol consumption levels in an adult primary care population. JAMA Network Open, 3(5), e204687. Available from: 10.1001/jamanetworkopen.2020.4687 32401315 PMC7221504

[acer15479-bib-0017] Suciu, A.M. , Crisan, D.A. , Procopet, B.D. , Radu, C.I. , Socaciu, C. , Tantau, M.V. et al. (2018) What's in metabolomics for alcoholic liver disease? Journal of Gastrointestinal and Liver Diseases, 27(1), 51–58. Available from: 10.15403/jgld.2014.1121.271.ald 29557415

[acer15479-bib-0018] Voutilainen, T. & Kärkkäinen, O. (2019) Changes in the human metabolome associated with alcohol use: a review. Alcohol and Alcoholism, 54(3), 225–234. Available from: 10.1093/alcalc/agz030 31087088

[acer15479-bib-0019] Xie, Y. & Xie, W. (2020) The role of sulfotransferases in liver diseases. Drug Metabolism and Disposition, 48(9), 742–749. Available from: 10.1124/dmd.120.000074 32587100 PMC7469250

[acer15479-bib-0020] Xu, R. , He, L. , Vatsalya, V. , Ma, X. , Kim, S. , Mueller, E.G. et al. (2023) Metabolomics analysis of urine from patients with alcohol‐associated liver disease reveals dysregulated caffeine metabolism. American Journal of Physiology. Gastrointestinal and Liver Physiology, 324(2), G142–G154. Available from: 10.1152/ajpgi.00228.2022 36513601 PMC9870580

[acer15479-bib-0021] Xu, R. , Vatsalya, V. , He, L. , Ma, X. , Feng, W. , McClain, C.J. et al. (2023) Altered urinary tryptophan metabolites in alcohol‐associated liver disease. Alcoholism, Clinical and Experimental Research, 47(9), 1665–1676. Available from: 10.1111/acer.15148 PMC10782820 37431708

[acer15479-bib-0022] Xu, Y.F. , Hao, Y.X. , Ma, L. , Zhang, M.H. , Niu, X.X. , Li, Y. et al. (2023) Difference and clinical value of metabolites in plasma and feces of patients with alcohol‐related liver cirrhosis. World Journal of Gastroenterology, 29(22), 3534–3547. Available from: 10.3748/wjg.v29.i22.3534 37389241 PMC10303510

[acer15479-bib-0023] Yang, Z. , Gao, H. , Ma, J. , Liang, N.A. , Liang, S.P. , Huda, N. et al. (2024) Unique urine and serum metabolomic signature in patients with excessive alcohol use: an exploratory study. Alcoholism, Clinical and Experimental Research, 48(8), 1519–1528. Available from: 10.1111/acer.15398 38951043

